# Stable Dried Catalase Particles Prepared by Electrospraying

**DOI:** 10.3390/nano12142484

**Published:** 2022-07-20

**Authors:** Corinna S. Schlosser, Steve Brocchini, Gareth R. Williams

**Affiliations:** UCL School of Pharmacy, University College London, 29-39 Brunswick Square, London WC1N 1AX, UK; corinna.schlosser.20@ucl.ac.uk (C.S.S.); s.brocchini@ucl.ac.uk (S.B.)

**Keywords:** electrospraying, proteins, bovine liver catalase, solid-state protein formulation, protein stability

## Abstract

Therapeutic proteins and peptides are clinically important, offering potency while reducing the potential for off-target effects. Research interest in developing therapeutic polypeptides has grown significantly during the last four decades. However, despite the growing research effort, maintaining the stability of polypeptides throughout their life cycle remains a challenge. Electrohydrodynamic (EHD) techniques have been widely explored for encapsulation and delivery of many biopharmaceuticals. In this work, we explored monoaxial electrospraying for encapsulation of bovine liver catalase, investigating the effects of the different components of the electrospraying solution on the integrity and bioactivity of the enzyme. The catalase was successfully encapsulated within polymeric particles made of polyvinylpyrrolidone (PVP), dextran, and polysucrose. The polysorbate 20 content within the electrospraying solution (50 mM citrate buffer, pH 5.4) affected the catalase loading—increasing the polysorbate 20 concentration to 500 μg/mL resulted in full protein encapsulation but did not prevent loss in activity. The addition of ethanol (20% *v/v*) to a fully aqueous solution improves the electrospraying process by reducing surface tension, without loss of catalase activity. The polymer type was shown to have the greatest impact on preserving catalase activity within the electrosprayed particles. When PVP was the carrier there was no loss in activity compared with fresh aqueous solutions of catalase. The optimum particles were obtained from a 20% *w/v* PVP or 30% *w/v* PVP-trehalose (1:1 *w/w*) solution. The addition of trehalose confers stability advantages to the catalase particles. When trehalose-PVP particles were stored at 5 °C, enzymatic activity was maintained over 3 months, whereas for the PVP-only analogue a 50% reduction in activity was seen. This demonstrates that processing catalase by monoaxial electrospraying can, under optimised conditions, result in stable polymeric particles with no loss of activity.

## 1. Introduction

Peptides and proteins (polypeptides) actively participate in a wide range of physiological processes, being for instance involved in the transport of molecules, catalysing biochemical reactions, providing intra- and extracellular scaffolding support and forming receptors and channels in membranes [1,2]. A broad range of pathological conditions (e.g., genetic, metabolic, inflammatory and oncological diseases) are caused by abnormalities in protein function or proteins present at aberrant concentrations [2,3]. Thus, it is not surprising that interest in polypeptide therapeutics has increased considerably over the past decades [3]. The use of polypeptides as therapeutics also displays a number of advantages compared to low molecular weight pharmaceuticals. Proteins can have a very high specificity for their intended target and thus off-target effects are reduced compared with low molecular weight actives [3].

However, proteins are kinetically labile molecules and damage to their tertiary structure can be caused upon exposure to a non-physiological environment or physicochemical stress (e.g., temperature, pH, agitation, shear) [4,5]. It is important to maintain the polypeptide structure and conformation throughout its life cycle (production, formulation, storage, and administration to a patient) [4,6]. It has been shown that solid-state protein formulations offer stability advantages over liquid systems, especially at ambient storage temperatures [7]. Solid-state formulation may avoid the requirement of a cold chain, where sub-ambient temperature is maintained between the manufacturer and the patient, thus reducing economic and logistical burdens [3,8]. Among the commercialised solid state protein products, the main drying method is lyophilisation, though occasionally spray-drying is used [9]. However, both processing methods come with their limitations; lyophilisation induces a range of freezing and drying stresses, whereas spray-drying often requires temperatures above 100 °C and risks heat denaturation of the polypeptide [7,9]. 

In recent years, electrospinning and electrospraying have been increasingly explored for the formulation of polypeptides, such as enzymes (lysozyme, superoxide dismutase), growth factors (platelet derived growth factor-BB, human-nerve-growth factor), hormones (insulin, leuprolide) and antibodies [10,11,12,13,14,15,16,17]. These electrohydrodynamic (EHD) processes employ an electric field to rapidly evaporate solvent and solidify a polymer solution, resulting in the production of fibres or particles in the nano-to micrometre range, and have been used to encapsulate proteins and other therapeutic agents within a polymer matrix [3].

The properties of the polymer solution determine whether fibres or particles will be produced. Fibres are obtained when chain entanglement is high, which is usually achieved with high molecular weight polymers or concentrated polymer solutions. Particles will result from lower molecular weight polymers and less-concentrated polymer solutions [18]. Electrospraying produces particles at ambient temperature, thus avoiding heat induced protein degradation (as observed in spray-drying), and generally results in high encapsulation efficiencies. Furthermore, the low feed rate and atomisation process lead to overall low shear stress [19]. Monoaxial or blend EHD is achieved from a single solution where all components are mixed, whereas with co-axial EHD, a two-needle spinneret allows the production of core–shell structures from two separated solutions. Thus, with co-axial EHD, biomolecules can be protected from potential solvent induced denaturation and loss of function minimised, whereas the blend process often leads to denaturation and loss in bioactivity due to the harsh environment (e.g., organic solvents) [20,21]. However, co-axial EHD is more complex compared with the blend process, making it more challenging to scale up [3,20]. Onyekuru et al. have shown that proteins such as alkaline phosphatase can be processed by EHD into fibres and particles without loss of activity, even for blend solutions [20]. In fact, the blend electrospraying process maintained the integrity of alkaline phosphatase, whereas the co-axial process resulted in partial loss of activity, which was attributed to the high voltage that was required for the process [20].

Catalase is a ubiquitous enzyme found in almost all aerobic organisms, and three protein families have evolved [22,23]. The most abundant are the heme-containing families, which include typical catalase and catalase-peroxidases. The third family, the manganese-catalases, represent a minor family present only in bacteria [22]. Bovine liver catalase (H_2_O_2_ oxidoreductase, EC 1.11.1.6) belongs to the family of typical (heme-containing) catalases and is a tetrameric enzyme with a molecular weight of 240 kDa, composed of 4 identical subunits of about 60 kDa [22,23,24]. The enzyme catalyses the conversion of hydrogen peroxide (H_2_O_2_) into water and oxygen, and by controlling the concentrations of H_2_O_2_ contributes to the proper functioning of the body’s defence system and cell protection [24]. H_2_O_2_ is involved in intracellular messaging, thereby mediating various cell functions; however, excessive hydrogen peroxide levels are harmful for almost all cell components [22,25]. Indeed, H_2_O_2_ is one of the most frequently occurring reactive oxygen species and thus is involved in many diseases associated with oxidative stress. It can affect cells distant from its site of origin, since it easily crosses biological membranes [22,24]. Catalase has therefore been proposed as an antioxidant in the treatment of various conditions such as radiation damage, inflammation, and oxidative stress [24]. Furthermore, catalase is employed in photodynamic therapy (PDT), where excitation of a photosensitiser generates radical oxygen species which subsequently lead to tissue destruction [26]. As catalase generates oxygen by decomposition of H_2_O_2_ it can combat the hypoxic microenvironment often found in solid tumors, which is a limiting factor in PDT [27]. Electrosprayed catalase formulations could thus be beneficial as a basis for therapeutics for a range of conditions.

The objectives of this work were to optimise production of stable catalase-loaded particles by electrospraying, and to gain a better understanding of the factors affecting the processing of proteins via this route. The impact that different components in the electrosprayed solutions have on maintaining the protein activity within the produced particles will be assessed.

## 2. Materials and Methods

### 2.1. Materials

Bovin serum albumin, catalase from bovine liver lyophilised powder (2000–5000 units/mg protein), ethanol, hydrogen peroxide solution (30% *w/w* in H_2_O), methanol, polyvinylpyrrolidone (55 kDa), potassium hydroxide, Purpald^®^, sodium chloride, sodium citrate tribasic dihydrate, and sodium phosphate monobasic monohydrate were supplied by Sigma-Aldrich (Steinheim, Germany). Citric acid, L-arginine monohydrochloride, dextran (~70 kDa), and 2,2,2-trifluoroethanol (>99%) were provided by Alfa Aesar (Heysham, UK). Ethylene diaminetetraacetic acid disodium salt dihydrate (EDTA), sodium phosphate dibasic dihydrate, aqueous formaldehyde solution (35 wt%) and hydrochloric acid (36.5–38% in water) were obtained from Honeywell (Seelze, Germany). Potassium periodate was purchased from Scientific Laboratory Suppliers Ltd. (Nottingham, UK). Ficoll PM70 (polysucrose 70) was obtained from Cytiva (Uppsala, Sweden). The Bradford 5× reagent was obtained from Serva (Heidelberg, Germany) and a microBCA protein assay kit was sourced from ThermoFisher (Rockford, IL, USA). Super refined Polysorbate 20^®^ and super refined Polysorbate 80^®^ were obtained from Croda (Goole, UK). α,α-Trehalose dihydrate was purchased from Pfanstiehl (Waukegan, IL, USA).

### 2.2. Characterisation of Catalase and Particles

The methods presented below were obtained after extensive method development and validation to account for interference of the various excipients. Suitable methods for protein quantification should also consider and be tested for potential interference from denaturation of the protein (see Supporting Information Figures S1 and S2 for full details). 

#### 2.2.1. Bradford Assay

The micro assay described by the manufacturer was adapted to microplates with the final volume being 250 μL per well; thus, 200 μL of protein sample was mixed with 50 μL of reagent. After a minimum of 5 min incubation at room temperature, the absorbance was measured at 470 and 595 nm using a SpectraMax M2e plate reader (Molecular Devices, LLC, San Jose, CA, USA). All samples were prepared in 100 mM phosphate buffer (pH 7.0). Calibration curves were prepared in the presence of the polymer and polysorbate at the concentrations found in the particles to account for interference form the excipients (see Figure S1). 

#### 2.2.2. microBCA

Experiments were conducted using the micro-BCA protein assay kit. As per the manufacturer’s instructions a working reagent was prepared by mixing reagent MA, reagent MB and reagent MC at a ratio of 25:24:1 *v/v/v*. Protein samples (150 μL) were mixed with 150 μL of the working reagent prior to incubation at 37 °C for two hours. The absorbance at 562 nm was read using a SpectraMax M2e plate reader (Molecular Devices, LLC, San Jose, CA, USA). Calibration curves were prepared in the presence of PVP or PVP-trehalose at the concentrations found in the particles to account for interference from the excipients (see Figure S1). 

#### 2.2.3. Size Exclusion Chromatography

Catalase was assayed by HPLC-UV with a Hewlett Packard LC system (1100 series, USA) consisting of a quaternary pump equipped with a diode-array detector (λ = 230 nm, λ = 280 nm). A suitable method for size exclusion chromatography was developed on a Zorbax-GF450 column (6 μm particle size, 9.4 μm × 250 mm; see Table S1 and Figure S3). The mobile phase consisted of a 50 mM sodium phosphate buffer (pH 6.4) containing 400 mM arginine hydrochloride. Catalase was eluted using an isocratic method at a flow rate of 1.2 mL/min, column temperature of 30 °C, and UV detection at 280 nm. The injection volume was 50 µL.

#### 2.2.4. Turbidity Assay

Catalase solutions (1 mg/mL) were prepared in phosphate buffer (pH 7.0) and citrate buffer (pH 5.4). Additionally, solutions of citrate buffer (pH 5.4) with polysorbate 20 and polysorbate 80 (1:1 to 10:1 *w/w* polysorbate-to-catalase ratio) were prepared. The turbidity assay was performed as described by Li et al., where the absorbance determined at 600 nm using a SpectraMax M2e plate reader (Molecular Devices, LLC, San Jose, CA, USA) is an indicator of turbidity [28].

#### 2.2.5. Catalase Activity Assay

The catalase activity in different solutions was assessed using a previously published protocol with minor modifications [27]. Catalase-containing solutions were diluted in sample buffer (25 mM sodium phosphate buffer, 1 mM EDTA, 0.1% *w/v* bovine serum albumin, pH 7.5) to a catalase concentration of 5–10 μg/mL. A reagent mix containing assay buffer (100 mM sodium phosphate buffer, pH 7.0), methanol and aqueous H_2_O_2_ (35.28 mM) was prepared at a 10:3:2 ratio *v*/*v*/*v*. To each well of a 96-well plate, 20 μL of sample was added prior to initiation of the reaction with 150 μL of the reagent mix. The plate was incubated covered and protected from light on a plate shaker at room temperature. After 20 min, the reaction was stopped by adding 30 μL of 10 M aqueous KOH solution, followed by the addition of 30 μL Purpald solution (6.25 mg/mL Purpald in 480 mM aqueous HCl). The plate was once again incubated covered and protected from light on a plate shaker at room temperature. After 10 min, 10 μL of KIO_4_ solution (18.75 mg/mL KIO_4_ in 470 mM aqueous KOH) was added to each well to oxidise and reveal the coloured product formed between formaldehyde and Purpald. The plate was incubated at room temperature for 5 min. Finally, 100 μL of the reaction mixture was retrieved from each well and transferred to a clean 96-well plate where absorbance was read at 540 nm using a SpectraMax M2e microplate reader (Molecular Devices, LLC, San Jose, CA, USA). Unless otherwise specified a calibration curve was run in parallel and each formulation was assayed in triplicate. 

To determine the residual catalase activity within the electrosprayed particles, the formulation was first dissolved in sample buffer (25 mM sodium phosphate buffer, 1 mM EDTA, 0.1% *w*/*v* bovine serum albumin, pH 7.5) to a catalase concentration of 10 μg/mL. The catalase assay was then conducted as described above. The residual activity was calculated as detailed in Equation (1):

(1) $$\mathrm{Residual}\;\mathrm{activity}=\frac{Activity\;within\;particles\;}{Activity\;of\;reference\;}\times 100$$

#### 2.2.6. Morphology and Size Distribution

Samples were directly sprayed onto aluminium foil. A small square (~1 × 1 cm) was cut from the foil and mounted onto an aluminium stub (TAAB Laboratories, Aldermaston, UK) with carbon-coated double-side adhesive tape, prior to sputter coating with gold for 60 s (10 nm gold layer) using a Quorum Q150RS sputter coater (Quorum Technologies, Laughton, UK). The coated samples were then analysed using a Phenom Benchtop SEM (ThermoFisher, Eindhoven, Netherlands) with applied voltage of 10 kV. The size of the particles was determined using the Image J software version 1.53c (National Institutes of Health, Bethesda, MD, USA). For each formulation the size of 100 particles from three different frames each was determined. The size distributions were plotted using Prism version 8.4.0 (GraphPad Software, San Diego, CA, USA). 

#### 2.2.7. Fourier Transform Infrared Spectroscopy (FTIR)

FTIR spectra were obtained using a Spectrum 100 spectrometer (Perkin Elmer, Waltham, MA, USA). The spectral data were analysed with the OriginPro software (OriginLab Corporation, Northampton, MA, USA). Data were collected over the wavenumber range from 650 to 4000 cm^−1^, with a resolution of 1 cm^−1^ and 8 scans per sample.

#### 2.2.8. X-ray Diffraction (XRD)

XRD patterns were obtained on a MiniFlex 600 diffractometer (Rigaku, Tokyo, Japan) supplied with Cu Kα radiation (λ = 1.5418 Å) at a voltage of 40 kV and current of 15 mA. Measurements were recorded over the 2 θ range of 3–40° at a scan speed of 5° min^−1^.

### 2.3. Pre-Formulation

#### 2.3.1. Ethanol Concentration

From a catalase stock (1 mg/mL) prepared in aqueous citrate buffer (50 mM, pH 5.4) samples containing 50 μg/mL of catalase were prepared at increasing ethanol concentrations (0–50% *v*/*v*). Each sample was analysed using the SEC method immediately after preparation (0 h), as well as after 1.5, 3, and 6 h. The chromatogram for catalase showed two peaks where peak 1 (RT = 5.4 min) is thought to be the tetramer and peak 2 (RT = 8.4 min) is the co-elution of dimer and monomer (see Supporting Information, Figure S3). The effect of ethanol concentration on the protein was assessed by comparing changes with the area under the curve of peaks 1 and 2 after short term storage.

#### 2.3.2. Surfactants

Polysorbate 20 and polysorbate 80 solutions were prepared at 0.1%, 0.5%, and 1% (*w*/*v*) in citrate buffer (50 mM, pH 5.4). Each solution was employed for the preparation of a 1 mg/mL catalase stock. From this stock a further dilution was made to obtain a final concentration of 50 μg/mL. Controls were prepared in 100 mM phosphate buffer (pH 7) at 1 mg/mL and 50 μg/mL of catalase. All samples were stored for a minimum of 6 h before diluting to 5 μg/mL in the sample buffer for the activity assay (see Section 2.2.5). The activity of catalase in each solvent was determined and compared with the activity after storage at pH 7. 

### 2.4. Preparation of Catalase Particles

Different catalase formulations were prepared, in order to assess the impact of each component on the enzyme. Unless otherwise specified the solvent was an aqueous: ethanol mixture (80:20 *v*/*v*) where the aqueous solvent was citrate buffer (50 mM, pH 5.4). All catalase particles were prepared at a theoretical loading of 1 μg of catalase per milligram of polymer. Formulations (1.0 mL) were loaded into 1 mL disposable plastic syringes (BD Plastipack, San Augustin del Guadalix, Spain) with care taken to ensure there was no air bubble formation. The syringe was mounted on a syringe pump (KDS100, Cole Parmer, Vernon Hills, IL, USA). A 20 G stainless steel needle (inner diameter 0.61 mm, Nordson EFD, Aylesbury, UK) was connected to the tip of the syringe. The needle was then attached to a high voltage DC power supply (HCP 35-35000, FuG Elektronik, Schechen, Germany). The grounded electrode was connected to a Teflon coated collector plate (16 × 20 cm) which was situated 16 cm from the tip of the needle. The solution was dispensed from the syringe at a constant rate of 0.2 mL/h and the voltage was optimised for each formulation (15–22 kV). All experiments were conducted under ambient conditions of 19–30 °C and 25–50% RH. All particles prepared were analysed for protein content by Bradford assay and for residual activity (see Section 2.2.1 and Section 2.2.5 respectively).

#### 2.4.1. Surfactant Concentration

Solutions for this set of experiments contained 10% (*w*/*v*) of dextran as the carrier polymer and 100 μg/mL of catalase. The polysorbate 20 content was varied between 0 and 500 μg/mL. Following the first experiment, polysorbate 20 was compared with polysorbate 80 at the highest surfactant concentration (500 μg/mL).

#### 2.4.2. Solvent Type

The solutions here contained 10% (*w*/*v*) of dextran as the carrier polymer, 500 μg/mL polysorbate 20, and 100 μg/mL of catalase. Solutions were prepared with trifluoroethanol in place of ethanol, or without using a second solvent (i.e., an 100% aqueous solution of 50 mM citrate buffer (pH 5.4)). Where trifluoroethanol was used, the concentration was maintained at 20% *v*/*v*. The effect of the solution pH was also explored by replacing the citrate buffer with a phosphate buffer (100 mM, pH 7.0).

#### 2.4.3. Polymer Type

All formulations were prepared at 10% (*w*/*v*) of a polymer, 500 μg/mL polysorbate 20, and 100 μg/mL of catalase. The polymers compared were dextran, polyvinylpyrrolidone, and polysucrose. Unless otherwise stated, the solvent system comprised 20% *v*/*v* EtOH in 50 mM citrate buffer (pH 5.4).

#### 2.4.4. Polymer Concentration

All formulations were prepared with polyvinylpyrrolidone as the carrier polymer. The polymer concentration was varied between 10 and 20% (*w*/*v*) while maintaining the catalase concentration at 1 μg/mg of polymer. Some formulations also contained 10–20% (*w*/*v*) of trehalose. The polysorbate concentration was kept at 500 μg/mL. Unless otherwise stated, the solvent system comprised 20% *v*/*v* EtOH in 50 mM citrate buffer (pH 5.4).

### 2.5. Stability Evaluation of the Electrosprayed Particles

Two different formulations of the optimised catalase particles were electrosprayed, collected and stored in sealed glass vials under three different conditions: 5 °C, room temperature (~21 °C), and 40 °C/75% relative humidity. Table 1 presents the composition of the two formulations that were evaluated. For each, residual activity and protein content by microBCA (Section 2.2.2) were determined. All experiments were conducted in triplicate and results were compared with unformulated catalase stored in the same conditions.

### 2.6. Statistical Analysis

Unless otherwise specified, experiments were conducted in triplicate (*n* = 3) and results are presented as mean ± standard deviation. Statistical analysis was performed using ANOVA for comparing > two methods or Student’s *t*-test for the comparison of two methods. When ANOVA showed significant differences, Tukey’s post hoc test was employed. Statistical significance was set at *p* < 0.05.

## 3. Results and Discussion

### 3.1. Pre-Formulation

#### 3.1.1. Ethanol Concentration

**Figure 1:**
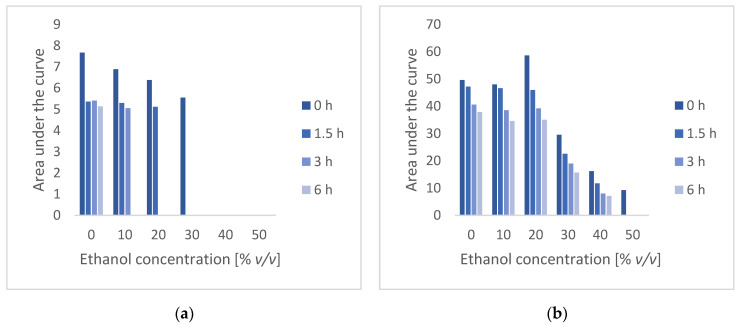
Area under the curve of catalase solutions (50 μg/mL), determined by SEC after storage at different ethanol concentrations (**a**) peak 1, (**b**) peak 2.

Electrospraying and electrospinning processes often use organic solvents which are known to cause protein denaturation [19,29]. Solvent concentration in water and hydrocarbon content of alcohols impact the extent of denaturation [30,31]. Attempting electrospraying of fully aqueous solutions is generally more challenging due to the high surface tension and electrical conductivity of water, which hinders formation of a stable Taylor cone-jet [19,32]. Furthermore, the boiling point of aqueous solutions leads to slow and sometimes incomplete evaporation which adds to the challenge of electrospraying fully aqueous solutions [19]. We thus first investigated to what extent ethanol could be used as a co-solvent with 50 mM citrate buffer (pH 5.4) The pH of the solution corresponds to the isoelectric point of catalase, ensuring that the overall charge of the enzyme is neutral and avoiding migration towards the surface (air-liquid interface) [15]. The effect of ethanol concentrations on the stability of catalase was assessed over a 6 h period, mimicking the duration of the electrospraying process. Figure 1 illustrates the changes in the area under the curve of the two catalase peaks in SEC after storage for 0–6 h at different ethanol concentrations. It is hypothesised that peak 1 (5.4 min) corresponds to the tetramer, whereas peak 2 (8.4 min) is a combination of dimer and monomer.

From Figure 1 it can be observed that the presence of ethanol does cause a reduction in the area under the curve for both SEC peaks immediately upon preparation, with the effect being particularly strong for ethanol concentrations of 30% *v*/*v* and above. Peak 1 decreases and disappears over time, especially for solutions containing a higher ethanol concentration. The area under the curve of peak 2 does also decrease over time, but up to a concentration of 20% *v*/*v* of ethanol no difference is observed compared with a purely aqueous system. These results concur with previous published work where the effect of ethanol as a cosolvent on whey protein isolate was assessed. In these experiments, solutions containing ≤ 20% ethanol showed low amounts of denatured whey protein isolate, whereas a drastic increase in denatured protein was observed at ≥ 20% ethanol [31]. Herskovits et al. studied solvent denaturation (aqueous-alcohol solutions) of a number of different proteins and found that for most alcohols (including ethanol), the denaturation rates were relatively rapid, suggesting that equilibrium should be obtained shortly after mixing [30]. Based on the above results, a 20% *v*/*v* ethanolic solution was used for the preparation of catalase particles by electrospraying. 

#### 3.1.2. Surfactant

Non-ionic surfactants are commonly added to protein formulations for their stabilising effect on macromolecules. In protein formulation they prevent adsorption of proteins to surfaces and reduce agitation-induced aggregation or denaturation [33,34].

**Figure 2:**
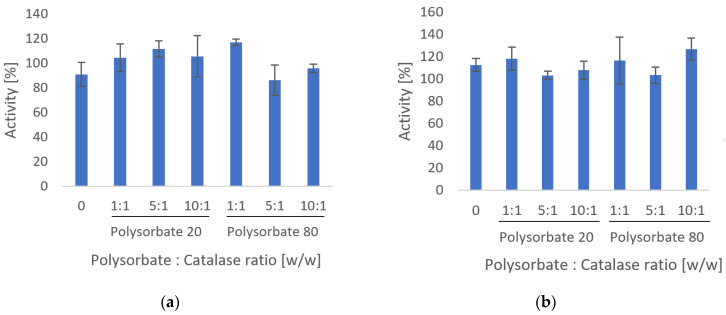
Catalase activity after storage at pH 5.4 in the presence of polysorbate 20 or polysorbate 80 at three different surfactant-to-catalase ratios (**a**) at 50 μg/mL catalase for 6 h, (**b**) at 1 mg/mL catalase for 24 h.

Polysorbate 20 and polysorbate 80 are the two most frequently used non-ionic surfactant excipients in protein formulations [35]. For a catalase solution in citrate buffer (50 mM, pH 5.4) turbidity and aggregation can be observed after <1 h (see Supporting Information, Figure S4). The effect of pH (pH 5.4 vs. pH 7) on catalase activity at two concentrations (50 μg/mL and 1 mg/mL) over a minimum of 6 h was assessed. Additionally, a range of polysorbate 20 and polysorbate 80 concentrations were added to solutions prepared at pH 5.4 to assess if the surfactants would impact activity (see Figure 2).

Figure 2 shows catalase activity after storage at pH 5.4, relative to the activity obtained from samples stored at pH 7. No difference in activity (one-way ANOVA, *p* > 0.05) can be observed between the different solutions, and all solutions displayed activity close to 100% compared to unprocessed catalase stored at pH 7. 

This experiment explored whether the use of surfactants could prevent protein aggregation in the electrosprayed samples. The pH of the protein solution is maintained at the isoelectric point of catalase (pI = 5.4), thus the net charge is zero and the protein is prone to aggregate due to reduced repulsion [36]. The addition of surfactants to the solution did not show any advantage in this regard, with no observable impact on turbidity or aggregation; however, this does not appear to affect the catalase activity. Because surfactants reduce the surface tension of aqueous solutions, which could facilitate the electrospraying process, and since no detrimental effect on catalase activity was observed in the presence of polysorbate 20 and polysorbate 80, they will be further explored [32].

### 3.2. Formulation Development

#### 3.2.1. Surfactant Concentration

Initial electrosprayed particles were prepared using dextran as the carrier polymer. Dextran is a non-reducing sugar mainly (~95%) composed of glucose units linked in position 1,6 with branches (~5%) linked 1,3 [37]. Non-reducing disaccharides (e.g., sucrose and trehalose) are commonly used as a cryoprotectant during freeze-drying of proteins [35]. As electrospraying is essentially a drying technique, we investigated if similar protecting properties could be obtained from a polysaccharide.

**Figure 3:**
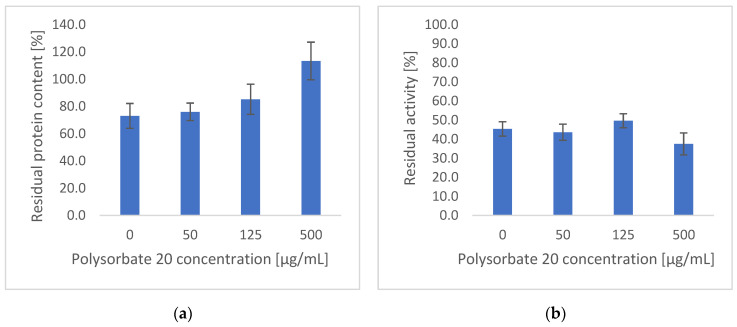
Protein content and activity for the catalase-dextran (1 μg/mg) particles electrosprayed from solutions containing 0–500 μg/mL of polysorbate 20. (**a**) catalase content, (**b**) catalase activity.

First, the impact of surfactants was assessed by adding increasing concentrations (0 to 500 μg/mL) of polysorbate 20 to the electrospraying solution, while maintaining all other components at a constant level. The effects of polysorbate concentration on protein content and residual catalase activity are shown in Figure 3.

All electrosprayed particles rapidly dissolve in phosphate buffer (used as solvent for these assays), thus allowing easy assessment of protein content and activity. An increase in polysorbate 20 concentration led to a higher protein content within the particles. Indeed, for particles prepared in the absence of surfactant, the protein content was 73.0 ± 9.1% compared to the theoretical value. This increases to 113.3 ± 13.8% at 500 μg/mL of surfactant, which is essentially a full recovery of the protein. The aqueous critical micellar concentration (CMC) of polysorbate 20 has been reported to be approximately 60 μg/mL [38], but CMC values have been shown to increase with addition of biopolymers (for instance, threefold in the presence of 20% resistant starch and fivefold in the presence of 20% maltodextrin) [32]. A slight but non-significant (one-way ANOVA, *p* > 0.05) increase in protein content in the particles is observed at 125 μg/mL polysorbate 20 compared with 0 and 50 μg/mL. Only the formulation containing 500 μg/mL polysorbate 20 shows a significant increase in protein content in the electrosprayed particles (one-way ANOVA, *p* < 0.05). Aqueous solutions of polypeptides exposed to solid surfaces can result in adsorption of the polypeptide onto the liquid–solid interface [33]. During the electrospraying process, proteins are exposed to surfaces during the preparation of the solutions (vials) but also during the electrospraying process (syringe and needle). A protein in a solution that is exposed to solid surfaces can adsorb onto the liquid–solid interface which can cause a loss in protein from the solution. This adsorption onto surfaces can be prevented by addition of excipients such as surfactants [33]. It is hypothesised that the incorporation of a surfactant such as polysorbate 20 increased protein content by reducing the nonspecific adsorption of catalase to exposed surfaces. Furthermore, it appears that concentrations above the CMC are required to observe any benefit. This could suggest that a surfactant monolayer is formed at the interface, similarly to literature reports where surfactant concentrations above the CMC have been shown to prevent protein aggregation [33].

In terms of residual catalase activity, between 38 and 50% of theoretical activity was detected for all samples; however, no significant difference (one-way ANOVA, *p* > 0.05) was observed. Thus, subsequent formulations were prepared containing 500 μg/mL of polysorbate to maximise protein encapsulation within the electrosprayed particles. 

In an additional experiment, the two polysorbates—20 and 80—were compared at 500 μg/mL in the electrospraying solution (see Supporting Information, Figure S5). While they did not show any significant difference in terms of catalase activity (~38%), the protein content within the polysorbate 80 particles was much lower (58.2 ± 7.1%) compared with polysorbate 20. All onward formulations hence contained polysorbate 20.

#### 3.2.2. Solvent

The remaining activity of catalase within the particles prepared above did not exceed 50% of the theoretical value. To assess if a change in solvent would maintain catalase stability during the electrospraying process, changes in the solvent were made. Ethanol (20% *v*/*v* in citrate buffer) was either replaced by trifluoroethanol (which has been shown to stabilise different peptides and proteins at low concentration) or the organic solvent was completely omitted (100% aqueous solvent used) [29,39]. In a separate experiment, the citrate buffer (50 mM, pH 5.4) was replaced by phosphate buffer (100 mM, pH 7.0) keeping 20% *v*/*v* ethanol. No significant difference (*t*-test, *p* > 0.05) in terms of protein content and residual activity were observed between the particles obtained at pH 5.4 and pH 7 (see Supporting Information, Figure S6). Furthermore, there was no advantage of removing ethanol from the electrospraying solution (100% aqueous solvent) but complete protein denaturation was observed, with no detectable catalase activity, when replacing for ethanol with trifluoroethanol (see Supporting Information, Figure S7). 

#### 3.2.3. Polymer Type

As previously mentioned, non-reducing disaccharides (e.g., sucrose and trehalose) are commonly used as a cryoprotectant during freeze-drying of proteins [35]. Initially in this work, a polysaccharide, dextran, was chosen as the carrier polymer to investigate if it could maintain protein activity during drying by electrospraying. This was, however, not completely successful, and the dried product only retained approximately 50% of its theoretical catalase activity. Thus, the impact of the carrier polymer on the catalase particles was assessed by replacing dextran with either polysucrose or polyvinylpyrrolidone (PVP). Polysucrose is another non-reducing polysaccharide analogous to dextran but where the repeat saccharide unit is glucose. The repeat unit in polysucrose is derived from sucrose linked by an epichlorhydrin-derived linkage. Polyvinylpyrrolidone is not a polysaccharide. It is a hydrophilic synthetic polymer frequently used in electrospun and electrosprayed products where fast dissolution is required [18].

**Figure 4:**
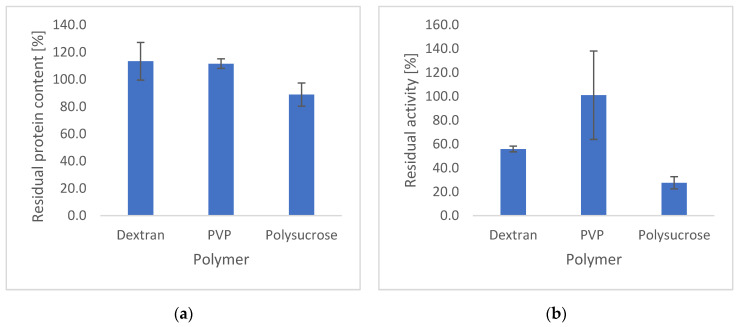
Protein content and activity for catalase-polymer particles (1 μg/mg) containing three different polymers (10% *w*/*v* in solution): (**a**) catalase content, (**b**) catalase activity.

The residual protein content and catalase activity within the three different catalase-polymer particles are shown in Figure 4.

The protein contents for the dextran and PVP formulations were close to 100%, whereas the protein content in the polysucrose sample was slightly lower (88.8 ± 8.5%). The residual catalase activity within PVP particles was determined to be 107.3 ± 26.1% and significantly higher (one-way ANOVA, *p* > 0.05) than the polysaccharide derived particles (activity for dextran 55.9 ± 2.4%, for polysucrose 27.6 ± 5.1%). The standard deviation for the PVP particles was however large, which could be attributed to instabilities observed during the process. Based on these results, PVP was used as the carrier polymer to prepare the optimal electrospun catalase particles. It was shown that the carrier polymer had the strongest influence on the protein stability as, contrary to the polysugars, PVP maintained the activity of the protein. For completeness, physical characterisation by FTIR and XRD of the dextran and polysucrose particles is shown in the Supporting Information, Figures S8 and S9, respectively. The IR spectra of the particles are dominated by the features of the polymer carrier, though show a shoulder or small peak at ca. 1635 cm^−1^ (likely corresponding to catalase Amide I). The XRD patterns show the particles to be amorphous, as are the starting materials.

### 3.3. Optimisation of the Electrospraying Conditions

The electrospraying experiments detailed above were conducted using a 10% *w*/*v* polymer solution. The electrospraying process, however, lacked stability—showing intermittent dripping from the needle—and thus required further optimisation. The results of a literature study investigating the feasibility of electrospraying fully aqueous solutions of bovine serum albumin suggested that a greater concentration of dissolved solids positively impacts the process, leading to a stable cone-jet mode and a dry powder bed [19]. This phenomenon was not only observed with an increase in the polymer content but also with increased protein concentrations [19]. The polymer content in solution is one of the factors determining if electrospraying or electrospinning will take place. Indeed, a higher polymer content allows for greater chain entanglement and results in electrospinning and a product in the form of fibres [18]. Thus, as an alternative to increasing the solid content in solution via the polymer concentration, trehalose—a disaccharide—was chosen to be added to some solutions. Trehalose is a low molecular weight compound added to protein formulations as a protectant during drying processes (e.g., freeze-drying) [33]. All stable processes (no dripping) in the abovementioned literature study contained 20% *w*/*v* of trehalose [19].

**Figure 5:**
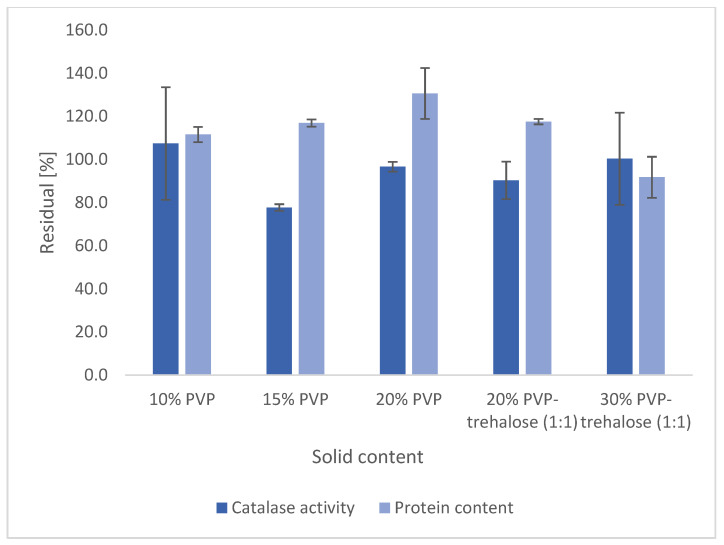
Protein content and activity in electrosprayed catalase-PVP (1 μg/mg) and catalase-PVP-trehalose (1 μg/mg) particles obtained from 10%, 15%, and 20% *w*/*v* PVP or 20% *w*/*v* and 30% PVP-trehalose (1:1) solutions.

Catalase particles were produced by electrospraying from PVP solutions of different concentrations (10–20% *w*/*v*) or PVP-trehalose (1:1 *w*/*w* ratio) solutions at different concentrations (total dissolved polymer/sugar content 20–40% *w*/*v*) while maintaining the protein-to-polymer ratio at a constant level (1 μg/mg). The other components of the spraying solutions were kept constant (500 μg/mL polysorbate 20 in 50 mM citrate buffer (pH 5.4) and 20% *v*/*v* ethanol). In Figure 5, the protein content and the residual activity of each formulation is presented.

Both protein content and residual activity remain close to 100% compared with the theoretical value of all five formulations, and no significant difference in activity was observed for any formulation (one-way ANOVA, *p* > 0.05). A 40% *w*/*v* PVP-trehalose solution was too concentrated for all components to solubilise and remain in solution, meaning this solution could not be investigated further. In this work, the solutions for electrospraying were typically prepared by dissolving and mixing all components apart from catalase and polysorbate 20. Catalase is then rehydrated with an aqueous polysorbate 20 solution which is added dropwise to the remaining components. However, at 40% *w*/*v* the addition of EtOH led to precipitation. When adding catalase directly to the polymer-trehalose solution, dissolution could not be achieved. 

#### 3.3.1. Particle Characterisation

**Figure 6:**
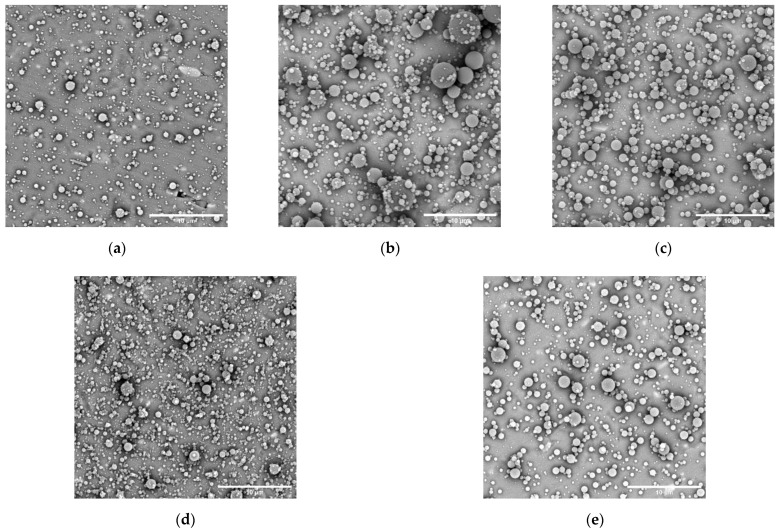
Scanning electron microscopy images of the electrosprayed catalase-PVP (1 μg/mg) particles prepared with solutions containing (*w*/*v*) (**a**) 10% PVP, (**b**) 15% PVP, (**c**) 20% PVP, (**d**) 20% PVP-trehalose, and (**e**) 30% PVP-trehalose. The scale bar represents 10 μm.

**Figure 7:**
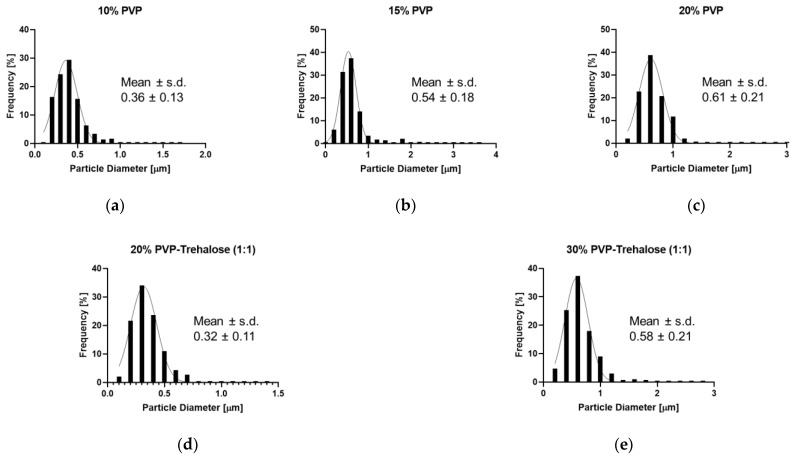
Particle size distribution of the electrosprayed catalase-PVP particles prepared from solutions containing (*w*/*v*) (**a**) 10% PVP, (**b**) 15% PVP, (**c**) 20% PVP, (**d**) 20% PVP-trehalose, and (**e**) 30% PVP-trehalose.

The morphology and particle size distribution of the catalase particles obtained from the five different PVP formulations are shown in Figure 6 and Figure 7, respectively. From the micrographs it can be observed that at 10% *w*/*v* PVP (Figure 6a) mainly round particles were obtained, but some long elliptical shapes were also present. This could be related to the process instability and the dripping that was observed. Such shapes are absent from the 15% and 20% *w*/*v* PVP formulations (Figure 6b,c, respectively), where only smooth particles were detected.

With regard to the PVP-trehalose formulations, the 20% *w*/*v* solution (Figure 6d) once again showed process instabilities and intermittent dripping throughout electrospraying, whereas the 30% *w*/*v* solutions (Figure 6e) led to a stable process. This could suggest that simply increasing the solid content was not sufficient to improve the electrospraying process, but rather that the polymer content had the most impact. Dilute solutions where polymer entanglement is low will produce particles with irregular morphology (see Figure 6a), whereas upon an increase in polymer concentration and thus entanglement sphere-like particles will be obtained [40]. As this phenomenon relates to polymer entanglement, it is evident that trehalose—a small molecular weight compound—cannot impact this.

A monomodal size distribution was observed for all formulations, with the mean diameter increasing for particles generated from more concentrated solutions. At 10% *w*/*v* PVP (Figure 7a) the mean diameter was 0.36 μm, which increases to 0.54 μm for the 15% PVP solution (Figure 7b) and to 0.61 μm for the particles prepared from 20% *w*/*v* PVP solutions (Figure 7c). For PVP-trehalose solutions the mean particle diameter was 0.32 μm and 0.58 μm at 20% and 30% *w*/*v* PVP-trehalose, respectively (Figure 7d,e). Comparing the mean particle diameter between all formulations, it appears that trehalose does not influence the particle size, as independent of the presence or absence of trehalose for the same PVP concentration similar particle sizes have been obtained. These trends are consistent with the literature, where it was found that it is the polymer concentration together with flow rate that determine the size of the electrosprayed particles [41].

#### 3.3.2. FTIR Spectroscopy

**Figure 8:**
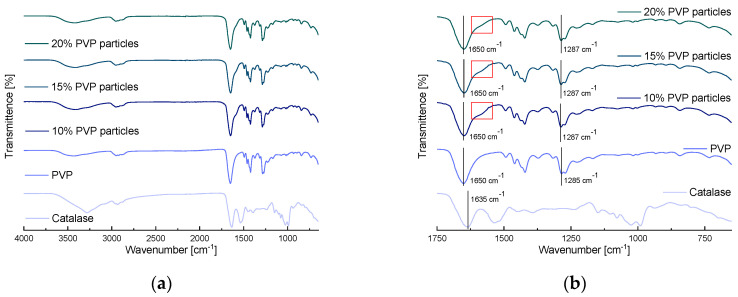

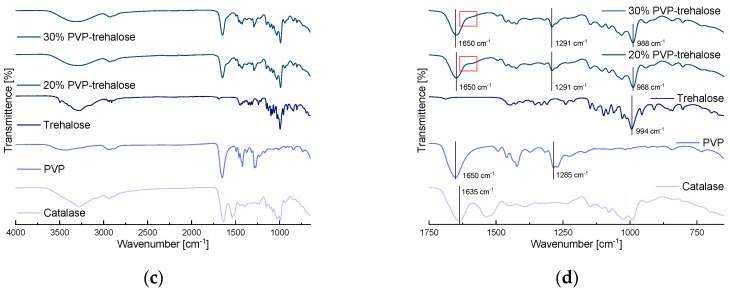
FTIR spectra of the raw materials and protein-loaded particles prepared from PVP solutions, showing (**a**) full spectrum and (**b**) enlargement of the 1750–650 cm^−1^ region, and PVP-trehalose solutions: (**c**) full spectrum and (**d**) enlargement of the 1750–650 cm^−1^ region.

The FTIR spectra of the raw materials and the different PVP and PVP-trehalose formulations are presented in Figure 8. 

PVP displays characteristic vibrations visible between 2840 cm^−1^ and 3000 cm^−1^ (C-H stretching), and at 1650 cm^−1^ (C=O stretching), 1421 cm^−1^ (C-H bending) and 1285 cm^−1^ (C-N stretching). A broad peak around 3000–3500 cm^−1^ is also observed, which originates from stretching vibrations of absorbed water [42]. For trehalose, vibrations are visible between 3200 and 3550 cm^−1^ (O-H stretching), 2840 and 3000 cm^−1^ (C-H stretching), 1300 and 1500 cm^−1^ (C-H bending) and at 994 cm^−1^ (C-O bending). Catalase displays the characteristic vibrations of a protein which include bands at 3300 cm^−1^ (amide A), 3100 cm^−1^ (amide B), 1635 cm^−1^ (amide I, originating from C=O stretching of the backbone carbonyl), 1550 cm^−1^ (amide II, originating from N-H bending with a contribution from C-N stretching), 1300 cm^−1^ (amide III, a weak signal arising from N-H bending and C-N stretching), and 735 cm^−1^ (amide IV) [35,43,44].

The IR spectra of the PVP-catalase particles are dominated by the features of the polymer carrier and, apart from a small bump to the right of the C=O stretch at 1600 cm^−1^ (red rectangle), the spectra are indistinguishable from the raw polymer. For the PVP-trehalose particles, similar observations are made; however, additional vibrational bands from trehalose, especially between 900 and 1500 cm^−1^, are visible. These results are not surprising as the protein loading within all formulations was very low (0.1% *w*/*w*), making it challenging to detect by IR. 

#### 3.3.3. XRD

**Figure 9:**
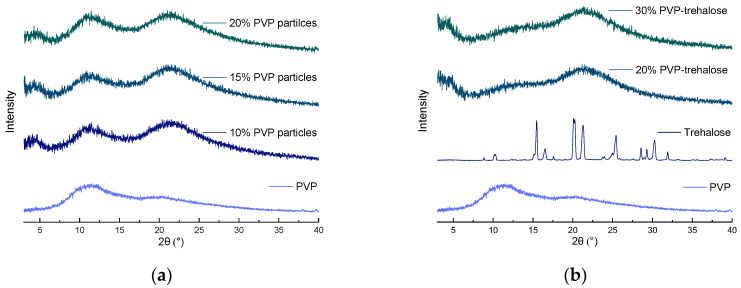
XRD diffraction patterns of the formulations and raw materials. (**a**) PVP particles, (**b**) PVP-trehalose particles.

XRD analysis provides information regarding the physical form of materials. The XRD patterns of the raw materials and the different electrosprayed PVP and PVP-trehalose particles are shown in Figure 9. Trehalose displays a series of sharp Bragg reflections which match with the crystalline dihydrate form, whereas PVP displays only a broad halo in its diffraction pattern (typical for amorphous materials) [45]. It can be observed that after electrospraying, trehalose Bragg reflections were absent in all the formulations, thus demonstrating their fully amorphous nature. This is expected as during the electospraying process all components were mixed on the molecular level, and due to the extremely rapid evaporation process (order of 10^−2^ s) there is insufficient time for molecular reorganisation and crystallisation [46].

For long-term stability of solid state protein formulations, it is generally desirable to maintain both the excipient and the protein in the amorphous form [47,48]. The amorphous form presents characteristics important for the stabilisation of biologics [49]. Indeed, in the amorphous form molecules are not organised in a crystal structure and thus are less constrained, allowing for stronger protein-excipient interactions via hydrogen bonds, whereas the recrystallisation of excipients results in phase separation, producing a crystalline phase and poor protein stability (as observed by Li et al. [49,50]). The latter authors examined the protection of calmodulin upon lyophilisation in the presence of different carbohydrates. Mannitol was the only excipient that recrystalised, which resulted in poor protein stability [50].

### 3.4. Catalase Stability in the Electrosprayed Particles

**Figure 10:**
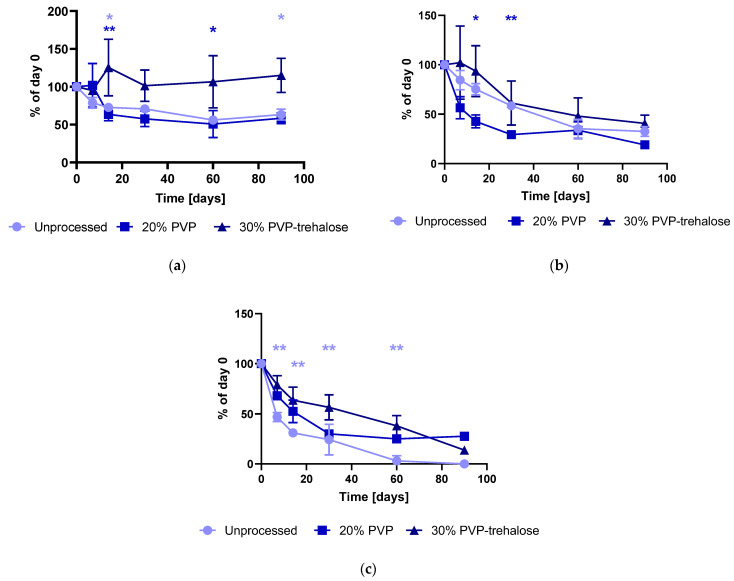
Residual activity based on measured protein content within the polymeric particles (1 μg/mg catalase:polymer) and unformulated catalase stored over 90 days at (**a**) 5 °C, (**b**) room temperature, and (**c**) 40 °C/75% RH. Where the 30% PVP-trehalose formulation is statistically significant different to either 20% PVP (dark blue asterisk) or unprocessed catalase (light blue asterisk) at a given time point, this has been indicated by (*) *p* < 0.05 and (**) *p* < 0.01.

Three batches of catalase particles were produced containing either PVP (20% *w*/*v* solution) or PVP-trehalose (30% *w*/*v* solution; 1:1 *w*/*w* PVP-trehalose) as the carrier. In both particles, the final catalase concentration was 1 μg of catalase per milligram of polymer. The catalase activity measured immediately after electrospraying was 73.9 ± 10.7% and 60.7 ± 9.5% for PVP and PVP-trehalose, respectively. The reduction in activity compared to the particles prepared in Section 3.3 could be due to the time it took to electrospray the required quantity of particles for the stability study. For the optimisation experiments, only a small quantity of particles was prepared (approximately 1 h–1.5 h of electrospraying), whereas for the stability batch particles were electrosprayed for 5 h (to yield 150–200 mg of particles). The stability was assessed by comparing the catalase activity (accounting for the protein content) within the particles to day 0 data and to unprocessed catalase stored under the same conditions (see Figure 10).

From Figure 10, it can be observed that apart from the 30% PVP-trehalose particles stored at 5 °C, the catalase activity within the particles decreased over time. Indeed, the enzymatic activity measured from PVP-trehalose samples stored at 5 °C averages approximately 100% over the 90 days storage. However, the experiment-to-experiment variability is high in these samples, which could contribute to the absence of significance compared to unprocessed catalase and the 20% PVP formulation. In fact, only on day 14 and day 90, was significant higher activity measured compared with unprocessed catalase, whereas at day 14 and day 60 the 30% PVP-trehalose particles had a significantly higher activity compared with 20% PVP.

When stored at room temperature (Figure 10b), the 30% PVP-trehalose formulation showed significant differences (*p* < 0.05) to PVP during the early time points (day 7 and day 14), as well as significantly higher activity (*p* < 0.01) compared to unprocessed catalase stored at 40 °C/75% RH between day 7 and day 60 (included) (Figure 10c). Particles exposed to 40 °C/75% RH storage conditions require a tight seal; otherwise, the particles crystalised onto the glass vials, as seen for two of the 20% PVP samples after 30 days and one of the 30% PVP-trehalose samples at day 90. It was not possible to quantify catalase content and activity within those samples, thus explaining the absence of replicates (s.d.) and statistical comparison for those samples.

It appears that the PVP-trehalose particles are maintaining catalase activity more effectively than the PVP-alone analogues. Sugars are frequently employed as stabilisers of proteins during lyophilisation and storage [51]. Their ability to intimately mix with the protein and the formation of hydrogen bonds appears to be a condition for successful stabilisation [51]. However, large and molecularly more rigid sugars (e.g., oligomers and polymers) are less efficient protein stabilisers compared to low molecular weight sugars (e.g., trehalose) [50,51]. This is attributed to steric hindrance with larger molecules, and thus the formation of fewer hydrogen bonds and potential phase separation, as has been observed for IgG lyophilised in the presence of different molecular weight sugars [51]. A similar trend in stability has been observed for keratinocyte growth factor-2 (KGF-2) lyophilised in the presence of sucrose, trehalose, and hydroxyethyl starch (HES) [52]. All lyophilised formulations of KGF-2 aggregated; however, a greater extent of aggregation was observed when increasing HES content. In a different study, Mensink et al. lyophilised IgG with sugars of different molecular weight (342 Da–70 kDa) and observed that the aggregation tendency was smaller for formulations containing the low molecular weight sugars [51].

Thus, comparing the size of trehalose (342 Da) and PVP (55 kDa), it is expected that the presence of trehalose allowed for more intimate mixing and resulted in the formation of more hydrogen bonds and better stabilisation compared with PVP-only formulations.

## 4. Conclusions

Catalase-loaded polymer-based particles were fabricated by monoaxial electrospraying. The composition of the solution used for spraying greatly affected the protein loading and bioactivity within the particles. A polysorbate 20 content of 500 μg/mL ensured catalase loadings close to 100%. The greatest impact on catalase activity, however, is obtained from the polymer carrier, where PVP particles fully maintained activity, dextran maintained approximately half of the initial activity and polysucrose approximately one third. The electrospray process was further optimised for the PVP particles by increasing the polymer content in solution from 10% to 20% *w*/*v*, which resulted in a stable process and particles with monomodal size distribution. The addition of trehalose appears to be advantageous with regard to catalase stability within the particles. PVP-trehalose particles prepared from a 30% *w*/*v* solution maintained the activity of catalase (at 5 °C storage) over the entire period of the stability study (90 days), and also led to increased stability at room temperature and 40 °C and 75% relative humidity. 

## Figures and Tables

**Table 1:** Composition of the catalase solutions prepared in 50 mM citrate buffer (pH = 5.4).

Formulation	PVP [*w*/*v*]	Trehalose [*w*/*v*]	Polysorbate 20 [μg/mL]	Catalase [μg/mL]	Ethanol [*v*/*v*]
20% PVP	20%	-	500	200	20%
(% *w*/*w* in the dried particles)	(99.7)	-	(0.3)	(0.1)	-
30% PVP trehalose	15%	15%	500	300	20%
(% *w*/*w* in the dried particles)	(49.85)	(49.85)	(0.2)	(0.1)	-

## Data Availability

Data are available on request from the authors.

## Supplementary Materials

The following supporting information can be downloaded at: https://www.mdpi.com/article/10.3390/nano12142484/s1, Figure S1. Catalase calibration curves in phosphate buffer, PVP (20 mg/mL), and PVP-trehalose (1:1 *w*/*w*) (20 mg/mL) added to the solvent prepared (a) with the Bradford reagent for protein quantification, (b) by microBCA. Figure S2. Protein quantification for native catalase standards and heat denatured standards in phosphate buffer using the (a) the Bradford assay, (b) microBCA. Figure S3. Chromatograms of catalase eluted at from the Zorbax-GF450 column (a) sample dissolved in phosphate buffer, (b) sample dissolved in 1% SDS. Figure S4. Absorbance at 600 nm of solutions prepared at pH 5.4, pH 5.4 containing polysorbate 20 or polysorbate 80 at different surfactant-to-catalase ratios (*w*/*w*), and at pH 7. Figure S5. Protein content and activity for the catalase-dextran (1 μg/mg) particles electrosprayed from solutions containing 500 μg/mL of polysorbate 20 or polysorbate 80: (a) catalase content, (b) catalase activity. Figure S6. Protein content and activity for the catalase-dextran (1 μg/mg) particles electrosprayed from solutions of pH 5.4 or pH 7.0. (a) catalase content, (b) catalase activity. Figure S7. Characteristics of the electrosprayed catalase-dextran particles in presence of three different solvents (a) catalase activity, (b) catalase content. Figure S8. FTIR spectra of the raw materials and protein-loaded particles, showing the (a) dextran formulations, (b) PVP formulations, and (c) polysucrose formulations. Figure S9. XRD pattern of the raw materials and protein-loaded particles, showing the (a) dextran formulations, (b) PVP formulations, and (c) polysucrose formulations, Table S1. Chromatographic conditions for catalase analysis [19,32,35,42,43,44,47,53].
