# Supplementary material for: Stable Dried Catalase Particles Prepared by Electrospraying
Source: Nanomaterials (Basel). 2022 Jul 20;12(14):2484. doi: 10.3390/nano12142484 (PMC9322511; doi:10.3390/nano12142484)
Supplement: Supplementary file 1 [file nanomaterials-12-02484-s001.zip › nanomaterials-1764974-supplementary.pdf]

Supporting Information

Corinna S. Schlosser [1], Steve Brocchini [1] and Gareth R. Williams [1,*]

[1] UCL School of Pharmacy, University College London, 29-39 Brunswick Square, London WC1N 1AX, UK
* Correspondence: g.williams@ucl.ac.uk

Protein quantification

The potential interference of the excipients with each of the quantification assays was evaluated for the different polymers and surfactants. The surfactants (100 µg/mL) and the polymer (20 mg/mL) were added separately to catalase standards and assays conducted as described in the respective methods sections.

The Bradford protein quantification assay is sensitive to polymer interference as well as to the presence of surfactants (polysorbate 20 and 80), whereas the microBCA assay was affected by the polymer presence but not by the surfactants. **Error! Reference source not found.** illustrates the impact of PVP and trehalose on the protein quantification assays. It was observed that the inclusion of the excipient in the calibration standards still allows for a linear relationship between the protein concentration and absorbance, but the gradient of the curve and intercept vary significantly.

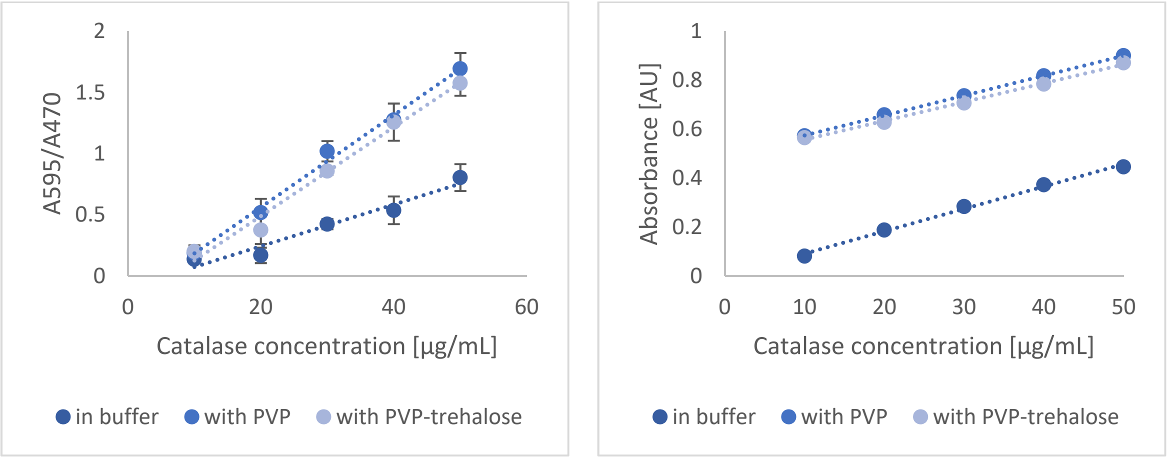

(**a**) (**b**)

Figure S1. Catalase calibration curves in phosphate buffer, PVP (20 mg/mL), and PVP-trehalose (1:1 *w*/*w*) (20 mg/mL) added to the solvent prepared (**a**) with the Bradford reagent for protein quantification, (**b**) by microBCA.

The impact of protein denaturation on the quantification assays was assessed by heat denaturing catalase (90 °C, 15 min). Different concentrations of catalase were prepared and denatured before conducting assays as described in Sections 2.2.1 and 2.2.2, and comparing the results to native catalase.

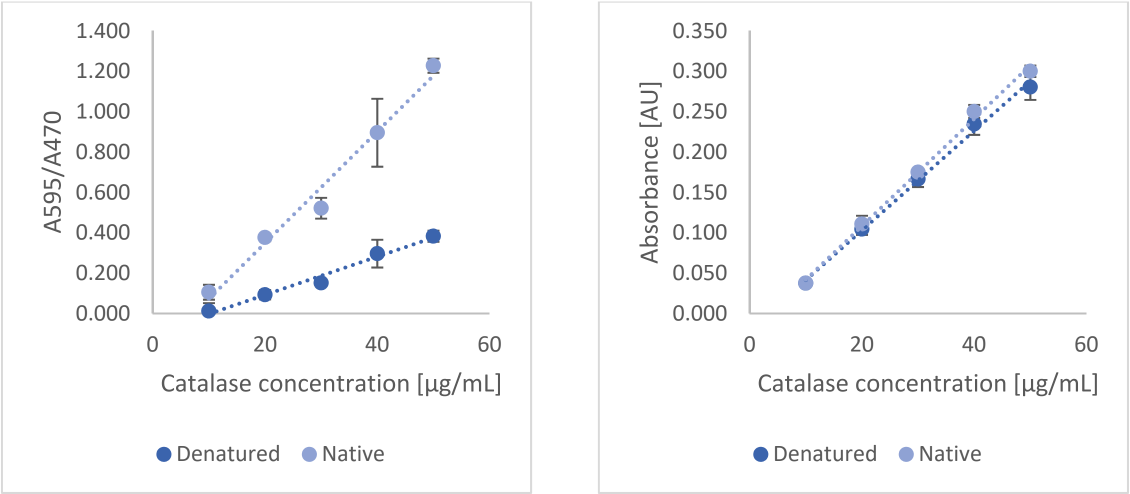

(**a**) (**b**)

Figure S2. Protein quantification for native catalase standards and heat denatured standards in phosphate buffer using the (**a**) the Bradford assay, (**b**) microBCA.

The experiment shows that protein quantification by Bradford assay is affected by protein denaturation (**Error! Reference source not found.**A). It is hypothesised that the Bradford dye binds to the protein surface and that heat denaturation led to a reduction in available surface, thus a reduction in the signal. The microBCA assay, however, does not show a significant difference between the absorbance values obtained from native and heat denatured protein standards (**Error! Reference source not found.**B). During the microBCA assay, Cu^{2+} is reduced to Cu^{+} via a redox reaction with specific amino acids. This reaction, however, is also triggered by peptide bonds, and thus the assay is less reliant on binding to specific amino acids [1]. The difference in size between Cu^{+} and the Coomassie dye could also contribute to the difference, with the Cu^{+} potentially penetrating the protein, thus being less reliant on the available surface compared to Coomassie.

Size exclusion chromatography of catalase

Catalase in phosphate buffer as well as denatured protein in 1% SDS was analysed by size exclusion chromatography (SEC) using a Zorbax-GF450 column with the conditions described in **Error! Reference source not found.**.

Table S1. Chromatographic conditions for catalase analysis.

Parameter	Sample A	Sample B
Mobile phase	50 mM phosphate buffer (pH 6.4) + 400 mM Arginine HCl	
Flow rate [mL/h]	1.2	
Sample concentration [mg/mL]	1	0.1
Sample diluent	Phosphate buffer	1% SDS
Injection volume [µL]	10	5
Column temperature	30 °C	
Wavelength [nm]	280 nm	
Run time	12 min	

The chromatograms obtained from native catalase (in phosphate buffer) and from the denatured protein (in 1% SDS) are presented in **Error! Reference source not found.**A and **Error! Reference source not found.**B, respectively. Both chromatograms present a peak at 5.4 min. In size exclusion chromatography molecules are separated based on their size, with larger molecules eluting faster than smaller ones. Considering that catalase has a quaternary structure (existing as a tetramer) it is hypothesised that this peak corresponds to the tetrameric form. Catalase in phosphate buffer presents a second peak at 8.4 min whereas when dissolved in a 1% SDS solution the second peak is observed at 9.0 min. Furthermore, a change in peak shape is observed. In **Error! Reference source not found.**A the peak shows a shoulder to the right whereas in **Error! Reference source not found.**B a small bump is observed at the left-hand side. It is hypothesised that in phosphate buffer the dimeric and monomeric forms are present and are almost co-eluting whereas in 1% SDS the protein is denatured leading to mainly monomeric catalase in solution, thus causing the shift in elution time.

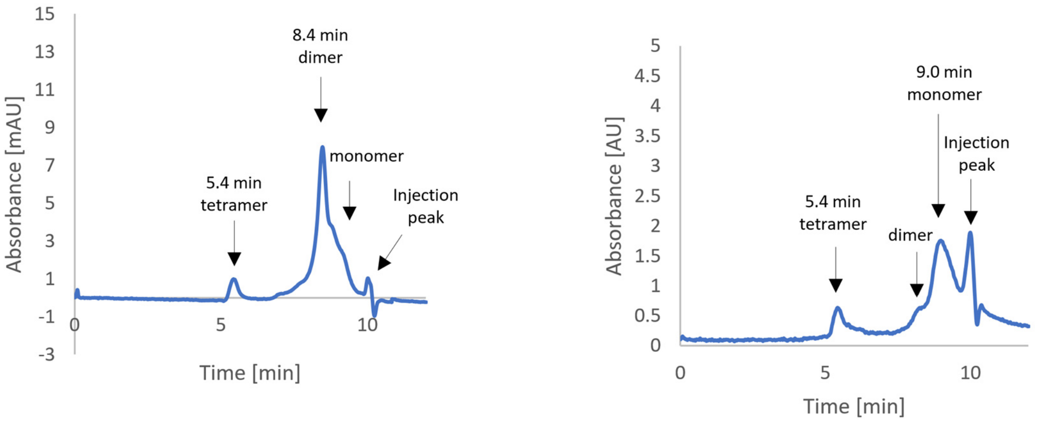

(**a**) (**b**)

Figure S3. Chromatograms of catalase eluted at from the Zorbax-GF450 column (**a**) sample dissolved in phosphate buffer, (**b**) sample dissolved in 1% SDS.

Turbidity assay

Error! Reference source not found. compares the absorbance measured at 0, 1, 2, 4, 6, and 8 h for different catalase solutions. It can be observed that apart from the solution at pH 7 the absorbance at 600 nm increased over the 8 h period. An increase in absorbance is caused by non-specific light scattering from aggregated particles causing turbidity of the solutions. The experiment thus confirmed the turbidity that was observed visually. The increase in turbidity at pH 5.4 (pI of catalase) likely originates from the formation of protein aggregates. The charge distribution close to or at the isoelectric point of a protein favours interaction between protein molecules, thus the formation of aggregates [2]. Furthermore, the addition of either polysorbate 20 or polysorbate 80 did not result in a reduction in absorbance and thus turbidity throughout the concentration range tested.

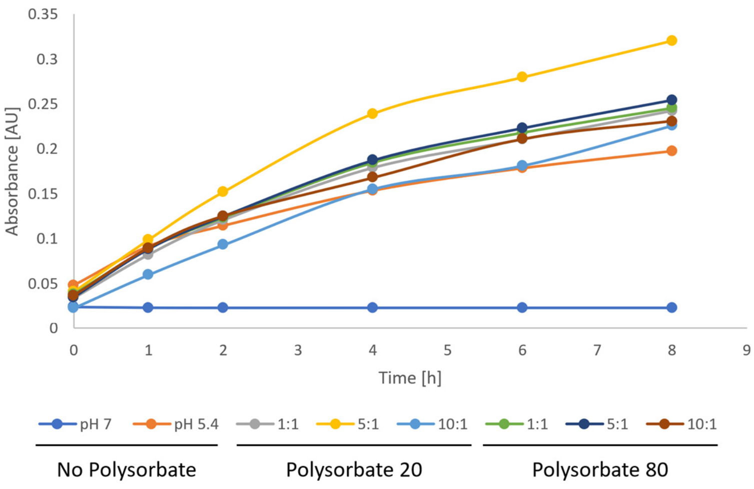

Figure S4. Absorbance at 600 nm of solutions prepared at pH 5.4, pH 5.4 containing polysorbate 20 or polysorbate 80 at different surfactant-to-catalase ratios (*w*/*w*), and at pH 7.

Polysorbate 20 vs. polysorbate 80

Data showing the influence of surfactant type (polysorbate 20 vs. polysorbate 80) on catalase activity after electrospraying are presented in **Error! Reference source not found.**.

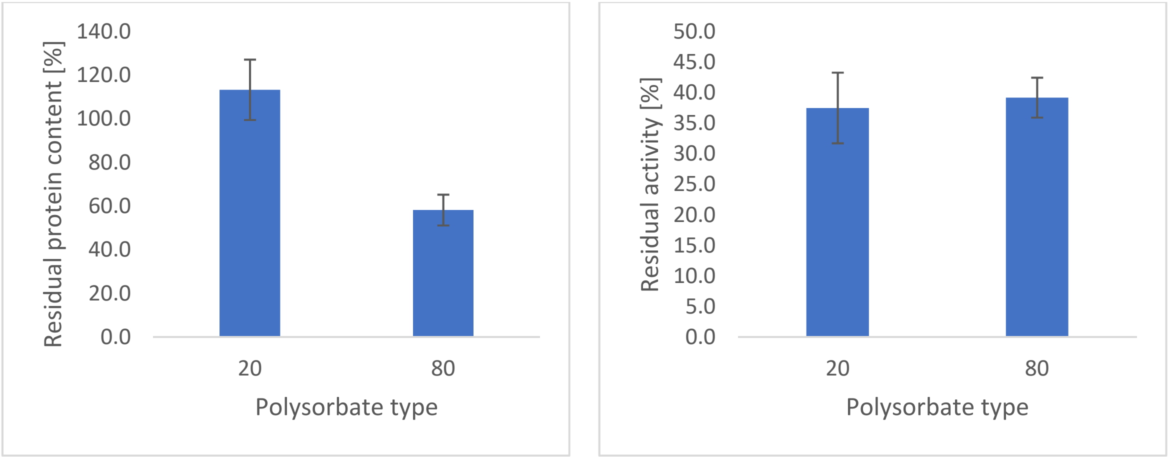

(**a**) (**b**)

Figure S5. Protein content and activity for the catalase-dextran (1 µg/mg) particles electrosprayed from solutions containing 500 µg/mL of polysorbate 20 or polysorbate 80: (**a**) catalase content, (**b**) catalase activity.

pH

Data showing the impact of the pH of the electrospraying solution on catalase activity after electrospraying are presented in **Error! Reference source not found.Error! Reference source not found.**.

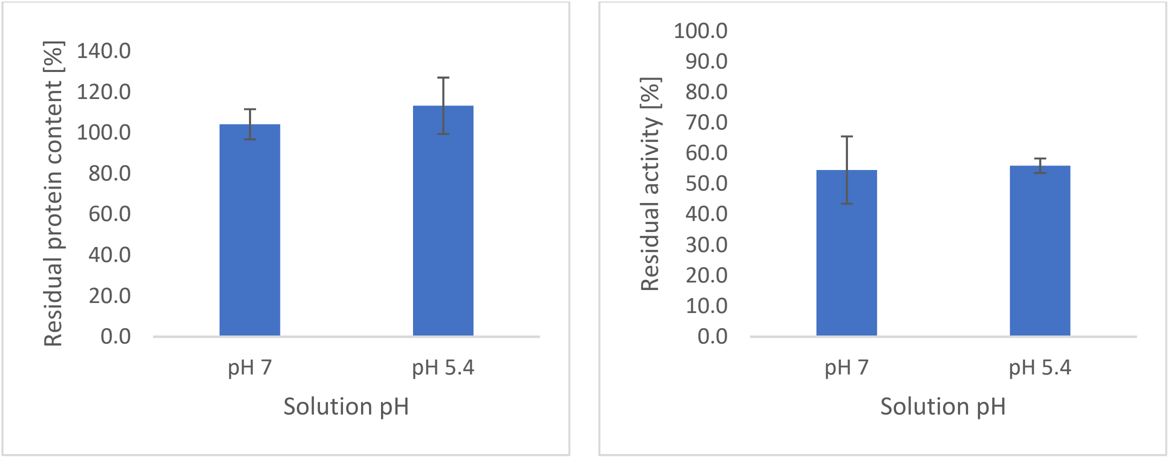

(**a**) (**b**)

Figure S6. Protein content and activity for the catalase-dextran (1 µg/mg) particles electrosprayed from solutions of pH 5.4 or pH 7.0. (**a**) catalase content, (**b**) catalase activity.

Solvent type

Data showing the influence of solvent on catalase activity after electrospraying are presented in **Error! Reference source not found.**.

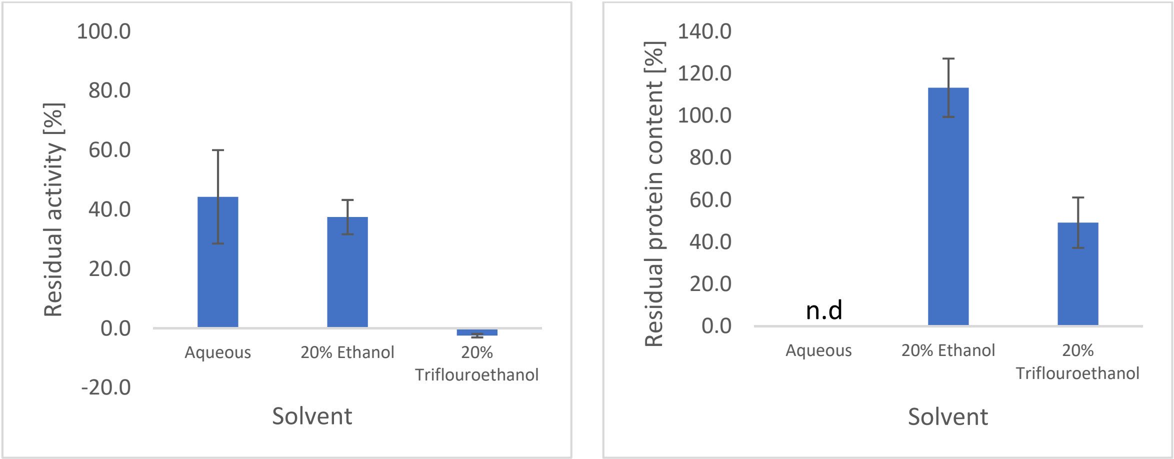

(**a**) (**b**)

Figure S7. Characteristics of the electrosprayed catalase-dextran particles in presence of three different solvents (**a**) catalase activity, (**b**) catalase content.

The residual catalase activity detected within the particles obtained from a fully aqueous solution was not significantly different (one-way ANOVA, $p > 0.05$) from those generated with the ethanolic solution. However, when trifluoroethanol was used as co-solvent, no activity was detected. The protein content within the particles generated from a fully aqueous solution could not be determined as the amount of sample recovered from the electrospraying process was not sufficient. This low yield obtained from fully aqueous solutions is related to the high surface tension of the aqueous solution, which prevents formation of stable jets [3]. The process could potentially be improved by further increasing the amount of surfactants or by increasing the solid content in solution [3,4]. However, as the use of ethanol as a co-solvent resulted in similar catalase activity, there would be no advantage in electrospraying 100% aqueous solutions. The protein content within particles electrosprayed from solutions containing triflouroethanol as co-solvent was determined to be 49.2 ± 12.0 %. The Bradford assay—used for protein quantification—is sensitive to protein denaturation (aggregation). Hence, the reduction in apparent

protein content could be due to denaturation (which would explain the low activity, **Error! Reference source not found.**A) rather than loss of protein during the process.

Polymer type

The FTIR spectra of the raw materials and the different polymeric particles are presented in **Error! Reference source not found.**.

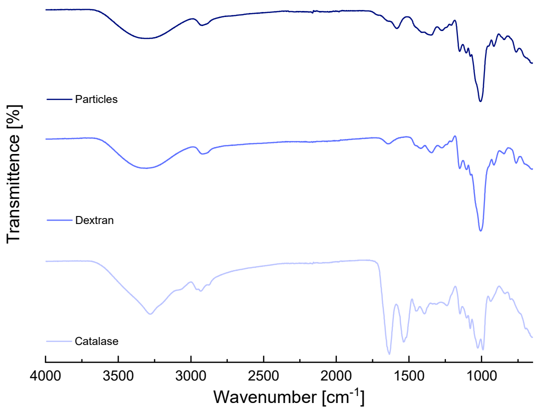

(**a**)

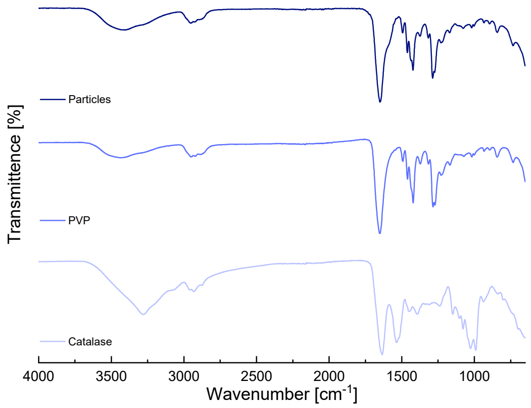

(**b**)

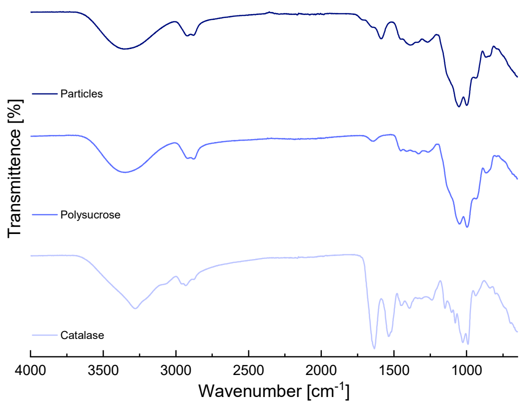

(**c**)

Figure S8. FTIR spectra of the raw materials and protein - loaded particles, showing the (**a**) dextran formulations, (**b**) PVP formulations, and (**c**) polysucrose formulations.

Dextran and polysucrose both present a broad peak around 3000 – 3500 cm^{-1}, which originates from O-H stretching vibrations of the hydroxyl groups. The characteristic vibrations of C-H stretching are observed between 2800 cm^{-1} to 3000 cm^{-1}. Differences between the two polymers are observed mainly in the fingerprint region (< 1500 cm^{-1}). PVP displays characteristic vibrations visible between 2840 cm^{-1} to 3000 cm^{-1} (C-H stretching), 1650 cm^{-1} (C=O stretching), 1421 cm^{-1} (C-H bending) and 1285 cm^{-1} (C-N stretching). The broad peak around 3000 – 3500 cm^{-1} originates from stretching vibrations of absorbed water [5].

Catalase displays the characteristic vibrations of a protein which include bands at 3300 cm^{-1} (amide A), 3100 cm^{-1} (amide B), 1635 cm^{-1} (amide I, originating from C=O stretching of the backbone carbonyl), 1550 cm^{-1} (amide II, originating from N-H bending with a contribution from C-N stretching), 1300 cm^{-1} (amide III, a weak signal arising from N-H bending and C-N stretching), and 735 cm^{-1} (amide IV) [6–8]. The IR spectra of the particles are dominated by the features of the polymer carrier. Indeed, apart from the small peak/bump at 1600 cm^{-1}, the spectra are indistinguishable from the raw polymer.

The XRD patterns of the raw materials and the different electrosprayed polymeric particles are shown in **Error! Reference source not found.**. All polymers as well as all the particles display only a broad halo in the diffraction pattern (typical for amorphous materials). This is expected given the amorphous nature of the starting materials.

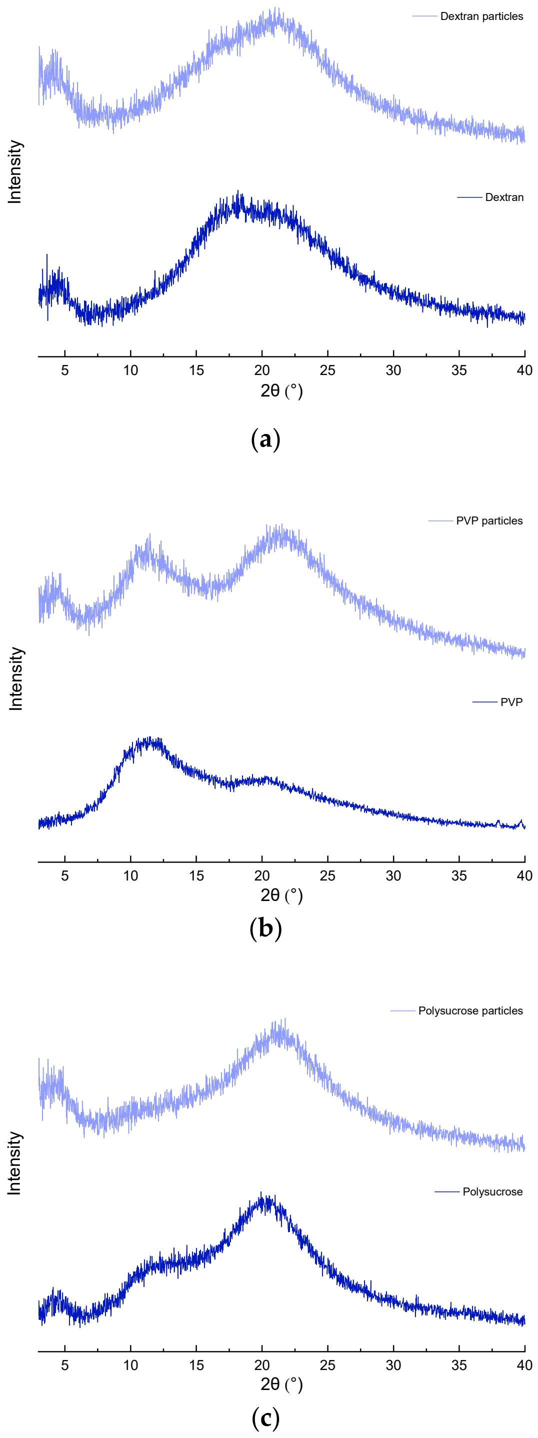

Figure S9. XRD pattern of the raw materials and protein - loaded particles, showing the (**a**) dextran formulations, (**b**) PVP formulations, and (**c**) polysucrose formulations.

References

1. Cortés-Ríos, J.; Zárate, A.M.; Figueroa, J.D.; Medina, J.; Fuentes-Lemus, E.; Rodríguez-Fernández, M.; Aliaga, M.; López-Alarcón, C. Protein Quantification by Bicinchoninic Acid (BCA) Assay Follows Complex Kinetics and Can Be Performed at Short Incubation Times. *Anal. Biochem.* **2020**, *608*, 113904, doi:10.1016/j.ab.2020.113904.
2. Chen, Y.; Mutukuri, T.T.; Wilson, N.E.; Zhou, Q. (Tony) Pharmaceutical Protein Solids: Drying Technology, Solid-State Characterization and Stability. *Adv. Drug Deliv. Rev.* **2021**, *172*, 211–233, doi:10.1016/j.addr.2021.02.016.
3. Pérez-Masiá, R.; Lagaron, J.M.; López-Rubio, A. Surfactant-Aided Electrospraying of Low Molecular Weight Carbohydrate Polymers from Aqueous Solutions. *Carbohydr. Polym.* **2014**, *101*, 249–255, doi:10.1016/j.carbpol.2013.09.032.
4. Batens, M.; Dewaele, L.; Massant, J.; Teodorescu, B.; Clasen, C.; Van den Mooter, G. Feasibility of Electrospraying Fully Aqueous Bovine Serum Albumin Solutions. *Eur J Pharm Biopharm* **2020**, *147*, 102–110, doi:10.1016/j.ejpb.2019.12.011.
5. Sharma, A.; Jain, C.P. Preparation and Characterization of Solid Dispersions of Carvedilol with PVP K30. *Res Pharm Sci* **2010**, *5*, 49–56.
6. Kamerzell, T.J.; Esfandiary, R.; Joshi, S.B.; Middaugh, C.R.; Volkin, D.B. Protein–Excipient Interactions: Mechanisms and Biophysical Characterization Applied to Protein Formulation Development. *Adv. Drug Deliv. Rev.* **2011**, *63*, 1118–1159, doi:10.1016/j.addr.2011.07.006.
7. Yang, H.; Yang, S.; Kong, J.; Dong, A.; Yu, S. Obtaining Information about Protein Secondary Structures in Aqueous Solution Using Fourier Transform IR Spectroscopy. *Nat Protoc* **2015**, *10*, 382–396, doi:10.1038/nprot.2015.024.
8. Haris, P.I.; Severcan, F. FTIR Spectroscopic Characterization of Protein Structure in Aqueous and Non-Aqueous Media. *J. Mol. Catal., N Enzym.* **1999**, *7*, 207–221, doi:10.1016/S1381-1177(99)00030-2.
